# Development of a 3-DOF Angle Sensor Based on a Single Laser Interference Probe

**DOI:** 10.3390/mi14122221

**Published:** 2023-12-10

**Authors:** Liang Yu, Xingyang Feng, Pengcheng Hu, Xionglei Lin, Tao Jing

**Affiliations:** Ultra-Precision Optoelectronic Instrument Engineering Center, School of Instrument Science and Engineering, Harbin Institute of Technology, Harbin 150080, China; liangyu@hit.edu.cn (L.Y.); 23s001005@stu.hit.edu.cn (X.F.); linxl@stu.hit.edu.cn (X.L.); jt20010527@163.com (T.J.)

**Keywords:** 3-DOF angle measurement, single beam interference, wavefront interference

## Abstract

The development of high-precision satellites has increased the demand for ultraprecision three-degrees-of-freedom (3-DOF) angle measurements for detecting structural deformation. The required instrument should simultaneously measure pitch, yaw, and roll angles using a single reference point. This paper proposes a 3-DOF angle measurement method based on the wavefront interference principle, and a mathematical model and its decoupling algorithm were built. Then, an angle-sensing probe with an extremely simple structure was designed and constructed. Finally, a series of experiments were performed to verify the method’s feasibility. The experiment results showed that the roll, pitch, and yaw measurement resolution of the probe was better than 10, 1, and 1 μrad, respectively, providing a high-performance 3-DOF angle measurement with a single probe. The short-term stabilities of roll, pitch, and yaw were better than 22, 1.7, and 2.0 μrad, respectively.

## 1. Introduction

Structural deformation of a satellite is a crucial factor that affects its performance. Such deformations reduce the aiming accuracy of space satellites and even affect the normal functioning of the space-based radar (SBR) [1,2,3]. With the improvement of star trackers and satellite attitude control accuracy, the requirements for the determination of the accuracy of structural deformation of some special satellites have reached the level of several microradians [4,5,6]. Structural deformation of satellites should be detected by simultaneous measurements of the changes in the pitch, yaw, and roll angles of the internal frame. Therefore, a highly integrated goniometer that provides a high resolution and stability is required for this purpose.

High-precision three-degrees-of-freedom (3-DOF) laser goniometers are widely used to simultaneously measure pitch, yaw, and roll angles. Currently, three main 3-DOF angle measurement methods are employed. The first and most commonly used is autocollimation for measuring three-dimensional angles [7,8]. SIOS measures the pitch and yaw angles using an autocollimator as the probe, while the roll angle is measured by a separate 1-DOF gradienter [9]. It has a high measurement resolution; however, there are two mutually perpendicular coordinate benchmarks in the goniometer corresponding to two separate probes. Therefore, the measurement precision is severely affected if the relative position of the two coordinate benchmarks changes. Such a goniometer requires regular calibration to ensure the correct installation position. Furthermore, such a goniometer occupies a large space and has a complex structure, thereby making its installation and adjustment difficult. Hsieh et al. constructed an orthogonal dual autocollimation right-angle measurement system using a beam splitter and a single laser light source to measure roll, pitch, and yaw angles with an accuracy of up to 0.04″ [10]. However, despite reducing the number of light sources, the probe still occupied a large space, and its working distance was short.

The second method is laser interferometry for angle measurement [11,12,13]. Keysight company arranged two parallel beam laser interferometers in mutually perpendicular directions. Herein, each probe uses multiple parallel beams to measure the pitch and yaw angles in the respective directions [14]. Therefore, the pitch, yaw, and roll angles can be calculated by summarizing the information from these two probes. Obviously, this method has the same issues as the SOIS’s scheme. In a multi-reflector interference system proposed by Ryoshu [15], the rotation angle is determined by measuring the optical path difference between two reflected beams, passing through a wedge prism and a spherical lens. The yaw and pitch angles are determined from the optical path difference of the corresponding two beams reflected by the spherical mirror. The measurement accuracy of the yaw, pitch, and roll angles is 2.35″, 1.67″, and 1.75″, respectively.

The third method comprises geometrical angle measurement and involves optical encoders and gratings [16,17,18]. The common drawback of geometrical methods is their poor measurement accuracy. Lee employed a single optical encoder to measure roll, pitch, and yaw angles with a single probe [19]. However, the measurement accuracy remained low (>7″ as compared to other methods). The internal structure of the probe was complex as seven beam splitters were used.

In summary, the existing 3-DOF goniometers possess the following disadvantages. High-precision goniometers comprise multiple discrete components. Their measurement standards are inconsistent, and the indicating values are prone to drift. The measurement accuracy of single-probe 3-DOF goniometers is also low, while the probe structure remains relatively complex. To address these disadvantages, we proposed a 3-DOF angle measurement method based on two interference fringes. Its core principle is based on the Twyman–Green interferometer improved by Molnar et al. [20,21]. This method was entitled “single beam interferometric imaging method for angle measurement”; however, just pitch and yaw angles were measured using one fringe [22].

In this paper, we proposed a novel 3-DOF angle measurement method based on the camera-type interferometer. The proposed angle-sensing probe comprises two sets of interferometers with a common nonpolarized beam splitter (NPBS). Herein, two interference patterns with fringes are formed owing to the special design of the interference structure. Such a pattern registered by a CMOS industrial camera contains information on the pitch, yaw, and roll of the measurement object. The 3-DOF angle is decoupled from this pattern by applying a high-precision algorithm. To demonstrate the feasibility of the proposed method, a dedicated goniometer was designed and tested for time stability, resolution, and measurement range.

## 2. Materials and Methods

### 2.1. Wavefront Interference Principle

**Figure 1:**
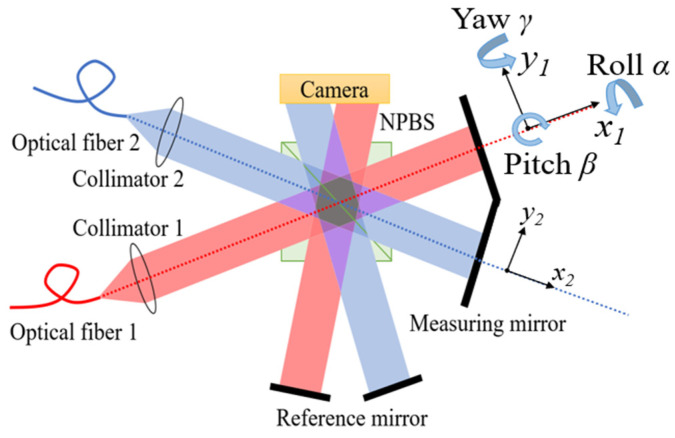
The schematic diagram of the 3-DOF angle measurement system.

A schematic of the 3-DOF angle measurement system is shown in Figure 1. There are two sets of homodyne interferometric systems in a diagram, whose optical path parts are distinguished by different colors: red and blue. The red part is defined as the primary interferometer (PI), and the blue part is denoted the auxiliary interferometer (AI). There is a nonzero tilt angle between the measuring mirrors of PI and AI. The right-handed coordinate systems x1-o1-y1 and x2-o2-y2 are established to clearly describe the relation between PI and AI. Therefore, the roll *α*, pitch *β*, and yaw *γ* denote the rotation around the *x*_1_-axis, *y*_1_-axis, and *z*_1_-axis, respectively.

In the case of PI, a frequency-stabilized laser is incident on the surface of NPBS after beam collimation and expansion through the collimators. The laser is divided into two beams by NPBS. The transmitted light beam is defined as a measurement light beam because it returns through a movable planar mirror. The reflected light beam is defined as a reference light beam because it returns through a fixed planar mirror. The two laser beams are combined again by NPBS, and interference fringes are formed by the overlapping measurement and reference beams due to the preset microwedge angle between the two planar mirrors. Then, the interference pattern is registered by a CMOS industrial camera. As PI and AI are completely similar in terms of interference structure, the structure of AI will not be introduced.

**Figure 2:**
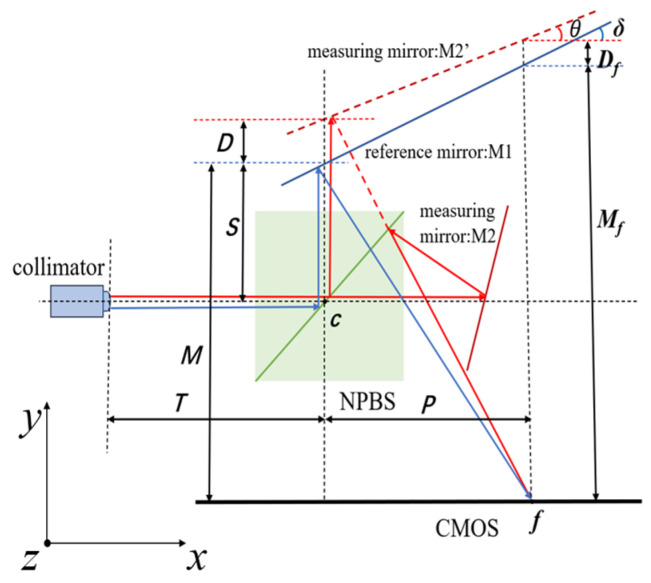
Geometric diagram of the single beam model.

Similarly, consider the angle measurement model of PI where the optical axes of laser beams coincide with the motion axis. This model is named the single-beam wavefront interference principle. As illustrated in Figure 2, a clear geometric relation of the optical path can be obtained easily in a two-dimensional projection of the measurement model. Herein, red arrows denote the measurement beam, and the blue arrows indicate the reference beam. *θ* is the inclination of the measuring mirror; *δ* is the preset small wedge angle of the reference mirror; *c* is the laser reflection point in the NPBS; *T* is the distance between c and the collimator surface; *S* is the distance between the reference mirror M1 and *c*; M2 and M2’ are the measurement mirror and its symmetrical projection, respectively; *D* is the distance between the laser reflection points in M2’ and M1; *f* is the point where the measurement and reference light ultimately form interference fringes on the camera CMOS; *P* is the horizontal distance between *f* and *c*; *M_f_* is the distance between M1 and *f*; and *D_f_* is the distance between M2’ and M1 in the direction of the *x*-axis.

According to the light trace method, the expressions for *D_f_* and *M_f_* can be derived:

(1) $$D_{f}=D-P\tan\delta +P\tan\theta$$

(2) $$M_{f}=M+P\tan\delta$$

**Figure 3:**
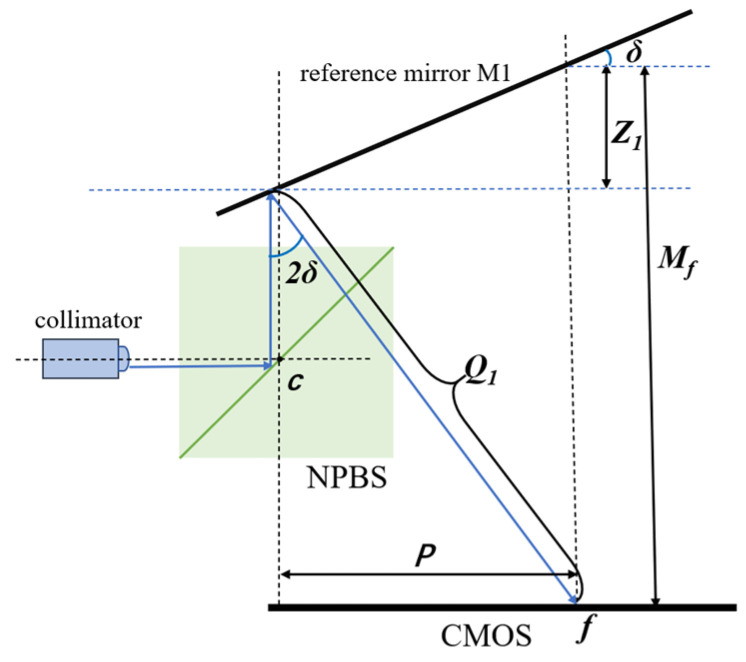
Reference light path. The description is in text.

As the fringes in the interference pattern are caused by the optical path difference (OPD) essentially, it is necessary to represent the measurement beam and reference beam paths separately (Figure 3), where *Q*_1_ is the optical path from the reference mirror M1 to the camera CMOS and *Z*_1_ is the distance between the theoretical and actual position of the laser beam reflected by M1.

The parameters *Q*_1_ and *Z*_1_ in the reference light path are used in the subsequent calculation of the OPD:

(3) $$Q_{1}=\frac{M_{f\;}\tan\delta \tan2\delta }{1+\tan\delta \tan2\delta }$$

(4) $$Z_{1}=\frac{M_{f}}{1+\tan\delta \tan2\delta \cos2\delta }$$

Similarly, for the measurement light path, *Q*_2_ and *Z*_2_ are represented by substituting *M_f_* with *M_f_* + *D_f_* in the mathematical model for the reference light as follows:

(5) $$Q_{2}=\frac{(M_{f\;}+D_{f})\tan\delta \tan2\delta }{1+\tan\delta \tan2\delta }$$

(6) $$Z_{2}=\frac{M_{f}+D_{f}}{1+\tan\delta \tan2\delta \cos2\delta },$$

Based on the geometric relationship of the above optical paths, the optical paths *L*_1_ and *L*_2_ of reference light and measurement light, respectively, can be expressed as:

(7) $$L_{1}=T+S+M_{f}-M+Q_{1}-Z_{1}$$

(8) $$L_{2}=T+S+M_{f}+D_{f}-M+Q_{2}-Z_{2}$$

Combining Equations (1)–(6), the OPD between the measuring and reference beams can be derived as follows:

(9) $$\Delta L=L_{1}-L_{2}=(D+P\tan\theta )\left(1+2\cos\theta \right)+M\left(\cos2\theta -\cos2\delta \right)-P\tan\delta \left(1+\cos2\delta \right)$$

Equation (9) is a complete expression of the OPD demonstrating the impact of the geometrical arrangement of each element in the interference structure on the OPD. Additionally, this equation reveals the mechanism of interference fringe formation. Moreover, the angular attitude of the measurement planar mirror directly affects the interference pattern. The influence of each angle on the interference pattern is discussed separately in Section 2.2 and Section 2.3 with the corresponding mathematical models.

### 2.2. Pitch and Yaw Measurement Principle

**Figure 4:**
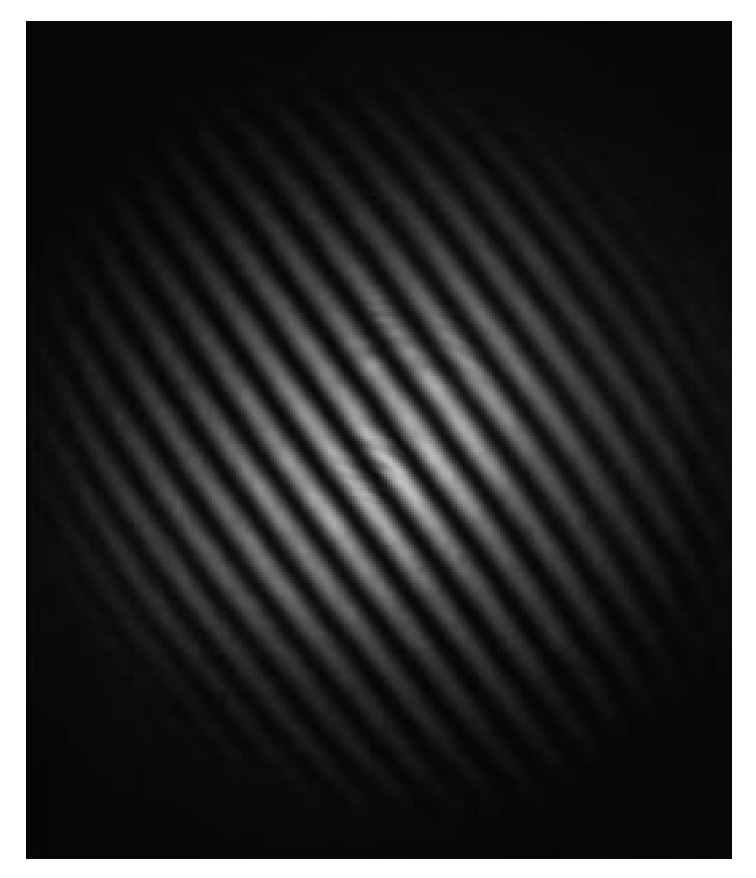
Single interference pattern captured by the CMOS camera.

In Section 2.1, the analysis of the optical path is simplified in a 2-dimensional plane. Actually, the laser beam emitted by the collimator is a cylinder spot with a certain radius. Due to the microwedge angle δ between the reference mirror and the optical axis, the OPD spatially oscillates, resulting in alternating light and dark fringes, as shown in Figure 4.

Equation (9) indicates that the OPD and interference fringes vary with change in the angle θ. OPD for the adjacent interference fringes satisfies the following relation:

(10) $$n(L_{1}-L_{2})=\lambda$$

where *n* is the air refractive index, and *λ* is the laser wavelength.

Thus, the horizontal fringe spacing $d_{x}$ can be represented as:

(11) $$d_{x}=\frac{\lambda }{n\left(\frac{2\tan\theta }{1+\tan^{2}\theta }-\frac{2\tan\delta }{1+\tan^{2}\delta }\right)}=\frac{\lambda }{n\left(\sin2\theta -\sin2\delta \right)}$$

As θ and δ are both small angles of a few milliradians, with the small angle linear approximation tanθ≈sinθ≈θ, the yaw *γ* can be expressed as:

(12) $$\gamma =\delta -\theta =\frac{\lambda }{2nd_{x}}$$

Defining the reciprocal of the fringe spacing $d_{x}$ as the fringe frequency $f_{x}$, Equation (12) can be expressed as:

(13) $$\gamma =\varepsilon_{x}=\frac{\lambda }{2n}\cdot f_{x}$$

Similarly, the pitch *β* can be expressed as:

(14) $$\beta =\varepsilon_{y}=\frac{\lambda }{2n}\cdot f_{y}$$

$\varepsilon_{x}$ and $\varepsilon_{y}$ are introduced as the parameters related to fringe frequency.

Equations (13) and (14) represent the linear decoupling models for the pitch and yaw angle, respectively. This demonstrates how the pitch and yaw can be directly decoupled by an interference pattern with fringes.

### 2.3. Roll Measurement Principle

As demonstrated in Section 2.2, one interference fringe can be used to decouple two angle degrees simultaneously. The pitch and yaw can be decoupled by using PI, but the roll angle still should be determined. Therefore, the second interferometer AI was introduced into the system to measure the roll. The AI is deflected at a yaw angle *φ* with respect to the PI, causing derivation of the AI optical axis from the motion axis of the measurement mirror. It will introduce the extra coupling error normally and needs to be avoided in most situations. However, analyzing the sources of coupling errors can calculate the roll angle. A precise coupling model is built in this section and used to measure a roll.

The coordinate transformation matrix between the AI and the PI can be easily derived based on the spatial geometric relationship. After defining *α* as a roll angle, the coordinate transformation matrix rotating around three axes can be represented as follows.

*R_x_* (roll angle), rotations around the *x*-axis:

(15) $$R_{x}\left(\alpha \right)=\left[\begin{array}{ccc} 1 & 0 & 0\\ 0 & \cos\alpha & -\sin\alpha \\ 0 & \sin\alpha & \cos\alpha \end{array}\right]$$

*R_y_* (pitch angle), rotations around the *y*-axis:

(16) $$R_{y}\left(\beta \right)=\left[\begin{array}{ccc} \cos\beta & 0 & \sin\beta \\ 0 & 1 & 0\\ -\sin\beta & 0 & \cos\beta \end{array}\right]$$

*R_z_* (yaw angle), rotations around the *z*-axis:

(17) $$R_{z}\left(\gamma \right)=\left[\begin{array}{ccc} \cos\gamma & -\sin\gamma & 0\\ \sin\gamma & \cos\gamma & 0\\ 0 & 0 & 1 \end{array}\right]$$

For the PI, in which the optical axis coincides with the motion direction, multiplying its initial vector $n=\left(\begin{array}{ccc} 1 & 0 & 0 \end{array}\right)$ by the rotation matrices by angles *α*, *β*, and *γ* around the *x*-axis, *y*-axis, and *z*-axis yields:

(18) $$n_{\alpha }^{\prime }=\left(\begin{array}{ccc} 1 & 0 & 0 \end{array}\right)$$

(19) $$n_{\beta }^{\prime }=\left(\begin{array}{ccc} \cos\beta & 0 & -\sin\beta \end{array}\right)$$

(20) $$n_{\gamma }^{\prime }=\left(\begin{array}{ccc} \cos\gamma & \sin\gamma & 0 \end{array}\right)$$

In this case, the laser vector does not depend on roll changes. This certifies that the roll cannot be measured through a single interference fringe. However, for the AI oriented at an angle *φ* to the motion direction, its initial vector can be expressed as:

(21) $$n=\left(\begin{array}{ccc} n_{x} & n_{y} & 0 \end{array}\right)=\left(\begin{array}{ccc} \cos\varphi & \sin\varphi & 0 \end{array}\right)$$

Then, after multiplication by rotation matrices (15)–(17), it yields:

(22) $$n_{\alpha }^{\prime }=\left(\begin{array}{ccc} n_{x} & n_{y}\cdot \cos\alpha & -n_{y}\cdot \sin\alpha \end{array}\right)$$

(23) $$n_{\beta }^{\prime }=\left(\begin{array}{ccc} n_{x}\cdot \cos\beta & n_{y} & -n_{x}\cdot \sin\beta \end{array}\right)$$

(24) $$n_{\gamma }^{\prime }=\left(\begin{array}{ccc} \cos\left(\gamma +\varphi \right) & \sin\left(\gamma +\varphi \right) & 0 \end{array}\right)$$

**Figure 5:**
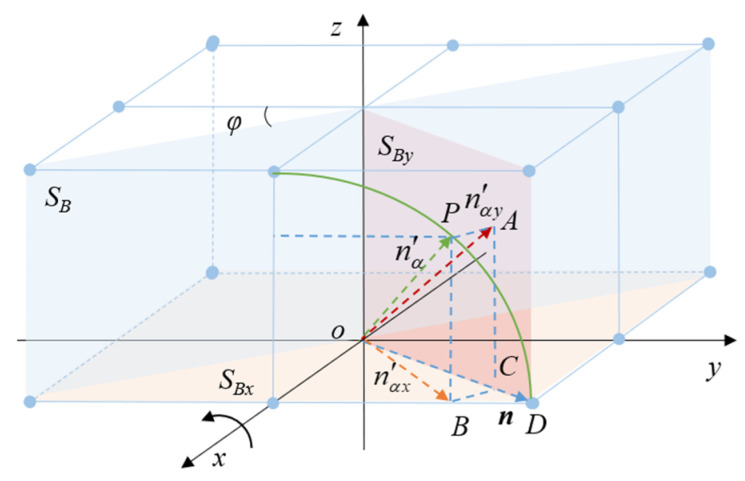
Vector decomposition relation.

Surprisingly, the roll will affect the reflected beam vector in this case. This means that the roll angle can be calculated according to the change in the reflected beam vector. As shown in Figure 5, $n_{\alpha }^{\prime }$ can be further divided into $n_{\alpha x}^{\prime }$ and $n_{\alpha y}^{\prime }$ along the coordinate axis. Moreover, *S_B_* is the mirror of AI. *S_B_*, *S_Bx_* and *S_By_* are perpendicular to each other. $n_{\alpha x}^{\prime }$ and $n_{\alpha y}^{\prime }$ are the projections of $n_{\alpha }^{\prime }$ on *S_Bx_* and *S_By_*, respectively. The green arc represents the trace of the light vector *n* when the roll angle changes.

The key geometrical relation in Figure 5 can be expressed as:

(25) $$\cos∠POB=\frac{\vec{OP}\cdot \vec{OB}}{\left|OP\right|\cdot \left|OB\right|}=\frac{{\vec{n_{\alpha }}}^{\prime }\cdot {\vec{n_{\alpha x}}}^{\prime }}{\left|{\vec{n_{\alpha }}}^{\prime }\right|\cdot \left|{\vec{n_{\alpha x}}}^{\prime }\right|}=\sqrt{n_{x}^{2}+n_{y}^{2}\cdot \cos^{2}\alpha }$$

(26) $$\cos∠DOB=\frac{\vec{n}\cdot {\vec{n_{\alpha x}}}^{\prime }}{\left|\vec{n}\right|\cdot \left|{\vec{n_{\alpha x}}}^{\prime }\right|}=\frac{n_{x}^{2}+n_{y}^{2}\cdot \cos\alpha }{\sqrt{n_{x}^{2}+n_{y}^{2}\cdot \cos^{2}\alpha }}$$

(27) $$\tan∠AOD=\frac{\left|AC\right|}{\left|OC\right|}=\frac{\left|AC\right|}{\left|OB\right|\cdot \cos∠DOB}=\frac{n_{y}\cdot \sin\alpha }{n_{x}^{2}+n_{y}^{2}\cdot \cos\alpha }$$

As the value *φ* is on the order of milliradians, some linear approximation of the relevant high-order terms can be considered, and Equation (27) will be simplified as:

(28) $$\alpha =\frac{1}{\varphi }\cdot {\varepsilon_{y}}^{\prime }$$

The formula above considers only the change in interference fringe caused by the roll change. In real situations, the interference fringe in the *y*-direction is determined not only by the roll but also depends on the pitch. Therefore, excluding this factor in the calculations along the *y*-direction is necessary. Considering the deflection angle *φ*, the pitch angle in the AI can be modified as follows:

(29) $$\beta_{1}=\frac{{\varepsilon_{y}}^{\prime }}{\cos\varphi }$$

**Figure 6:**
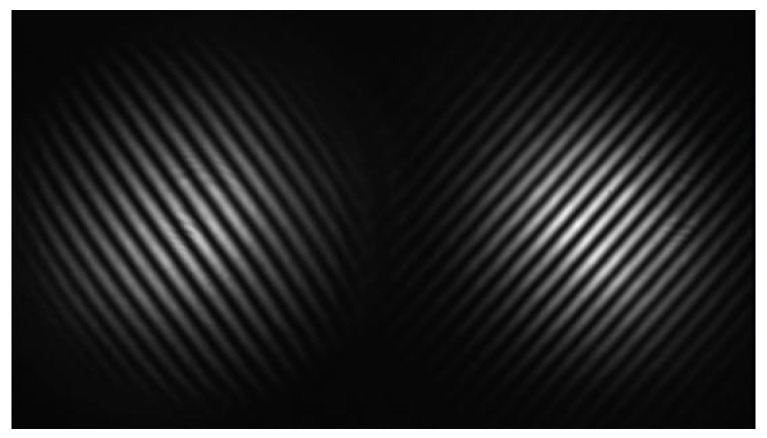
Two interference patterns captured simultaneously by the CMOS camera.

The captured image (Figure 6) comprises two interference patterns when introducing the AI.

The angles of the PI and AI systems vary in the same direction when the pitch changes. Conversely, the value of $\varepsilon_{y}$ and ${\varepsilon_{y}}^{\prime }$ vary in the opposite directions when the roll changes. Therefore, the pitch and roll can be calculated from the sum and difference of the fringe frequencies on the left and right spots, respectively.

In summary, Equations (13), (28) and (29) constitute the basic decoupling model of the 3-DOF angle, as proposed in this paper. Such a decoupling method can be considered to be linear at a small angle range and can be easily implemented. Additionally, the proposed 3-DOF angle measurement method can be modified when registering the angle change:

(30) $$\Delta \gamma =\Delta \varepsilon_{x}$$

(31) $$\Delta \beta =\frac{1}{2}\cdot (\Delta \varepsilon_{y}+\frac{\Delta {\varepsilon_{y}}^{\prime }}{\cos\varphi })$$

(32) $$\Delta \alpha =\frac{1}{2\theta }\cdot (\Delta \varepsilon_{y}-\frac{\Delta {\varepsilon_{y}}^{\prime }}{\cos\varphi }),$$

where Δ represents the angle change. Notably, Δ*ε* and Δ*ε*′ may be both positive and negative. Therefore, the fringe directions of the two interference light spots may differ. The pattern collected by a single camera should be decomposed with a discrete Fourier transform (DFT)-based pattern-decoupling algorithm to determine the fringe frequency of two fringes, $\varepsilon_{x}$ and $\varepsilon_{y}$.

### 2.4. Basic Decoupling Algorithm

**Figure 7:**
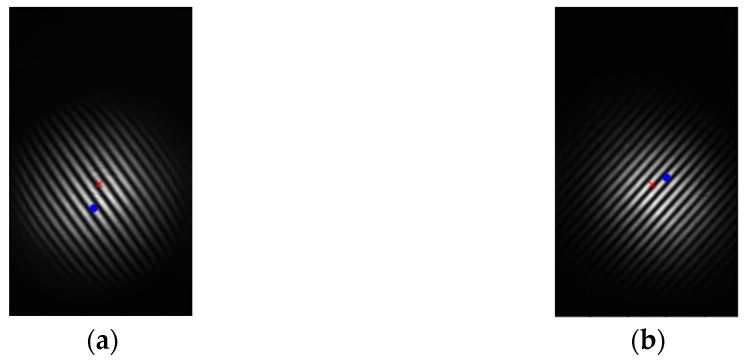
Spots after pattern segmentation. (**a**) Left spot, (**b**) Right spot.

The interference pattern after segmentation and determining the center of the light spot is shown in Figure 7, where “×” and “•” represent the center of the fringe and the brightest light point, respectively, as recognized by the program.

The geometric center of the light spot does not coincide with the brightest area. The main reason is that there is distortion after the laser light is emitted by the collimator because the surface of the planar mirror is not flat enough, and there is a “ghost” reflection inside the system.

The center point coordinates of the fringes were adopted to determine the longitudinal and transverse fringe frequencies on the two interference patterns. A DFT-based algorithm was performed on the horizontal and vertical fringes of each pattern to obtain the amplitude–frequency characteristic curve. Therefore, the frequency characteristic function of each vertical projection is represented as:

(33) $$X\left(m\right)=\overset{N-1}{\underset{n=0}{\sum }}x\left(n\right)e^{-j\frac{2\pi nm}{N}}$$

Here, *N* is the vector length, and *X*(*m*) is the complex frequency domain function after DFT. Then, the amplitude–frequency characteristic function can be expressed as the modulus of *X*(*m*):

(34) $$\left|X\left(m\right)\right|=\sqrt{X_{re}^{2}\left(m\right)+X_{im}^{2}\left(m\right)}$$

The subscript *re* and *im* represent the real part and the imaginary part, respectively. The frequency of the interference fringe is the distance between maximum amplitude points in the frequency characteristic curve. As the amplitude–frequency characteristic curve after DFT is symmetric around *π*, denoting *m*_1_ as the maximum value of |X(m)|, the fringe frequency can be expressed according to the conversion relation between the frequency domain and time domain in the DFT as follows:

(35) $$f=\frac{m_{1}}{N_{pixel}\cdot l},$$

where *N_pixel_* is the number of vertical/horizontal pixels of the camera, and *l* is the CMOS length.

## 3. Results

**Figure 8:**
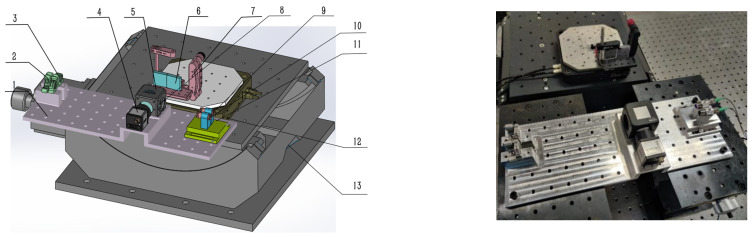
Mechanical model (**left**) and the external view (**right**) of the probe.

To verify the feasibility of the proposed angle measurement model, a 3-DOF angle-measuring probe was designed, as shown in Figure 8. Here, 1 is the measuring probe base; 2 and 3 are the reference reflector mirrors; 4 is the industrial camera; 5 and 8 are the NPBS frame and its prism; 6 is the measuring mirror glued with two rectangular mirrors; 7 and 11 are the regulators; 9 and 10 are the laser collimators; 12 and 13 are the combinatorial stage.

**Figure 9:**
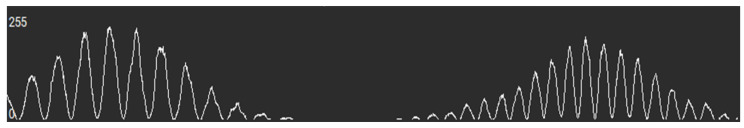
Distribution of light intensity registered by the camera.

The overall dimensions of the probe are 350 × 125 × 55 mm. The camera is a CMOS area scan industrial camera (U3-3280CP-M-GL, IDS) with a sampling rate from 0.5 to 36 fps and a 2448 × 2048 resolution. The light source is a semiconductor laser with a wavelength of 632.8 nm. To equally distribute the beam emitted by the laser between two collimators, 1 × 2 single-mode fiber couplers (SMC-635-50-FAU, LBTEK) with a light intensity ratio of 50/50 were used. A fixed aspherical fiber collimator with a waist diameter of 4 mm (AFC633-2.1-APC, LBTEK) was used to expand the laser beam. As the bit depth is set to 8 bits (0–255), extracting the horizontal fringes of the center light intensity in the interference pattern yields the following (Figure 9).

The two light spots were completely separate without any superposition in Figure 9. This means that the two interference fringes can be analyzed individually. The measurement mirror was fixed on a combinatorial stage. A high-precision nanopositioning system with 6 degrees of freedom (P-562.6CD, PIMar) with a minimum closed-loop tilt resolution of 0.1 μrad was used to perform yaw and pitch motion. A motorized goniometric cradles stages (MGC105, BOCIC) with the minimum incline angle of 10 μrad was used to perform the roll motion. Furthermore, the whole probe was placed on an air-floating damping platform to exclude the vibration impact. A number of tests were conducted on the performance of the probe, as described below.

### 3.1. Stability Test

**Figure 10:**
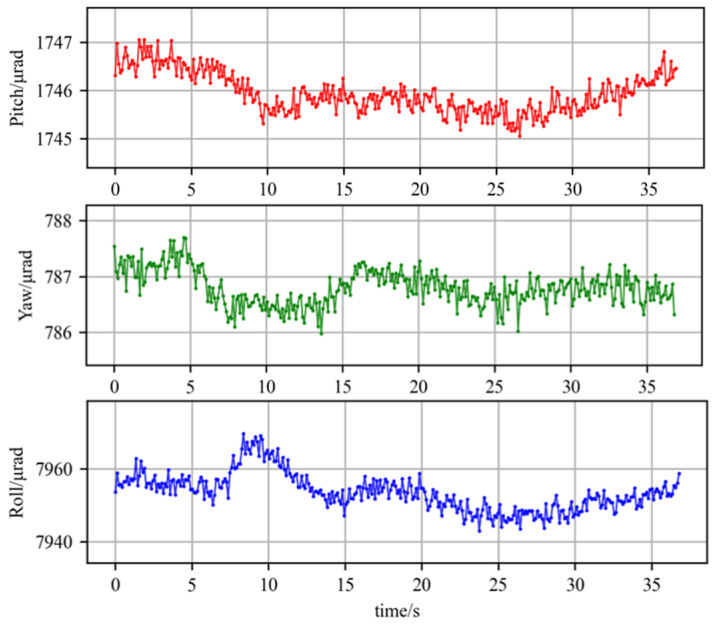
The short-term stability test.

Before the performance testing, the stability of the probe and the measurement environment was tested. First, a short-term stability test was conducted and the static results were collected for 35 s, as demonstrated in Figure 10.

The curve in Figure 10 shows that there is both high- and low-frequency noise during measurement. The amplitude of high-frequency noise is less than 1 μrad. It was caused by the fluctuations of the laser beam’s wavelength and intensity, background noise of the camera CMOS, and external vibration. This noise is similar to white noise and can be removed by a filtering algorithm. Another low-frequency noise had an amplitude of 1–2 μrad. It was caused by the drift of laser wavelength [23], the change in its optical path caused by the change in the geometric model geometry, and the change in the air refractive index due to heating [24,25].

The short-term stability results indicate that the roll, pitch, and yaw angles fluctuate within 22 μrad, 2 μrad, and 1.7 μrad, respectively, which are lower than those in other methods of measurements with a single probe [13].

**Figure 11:**
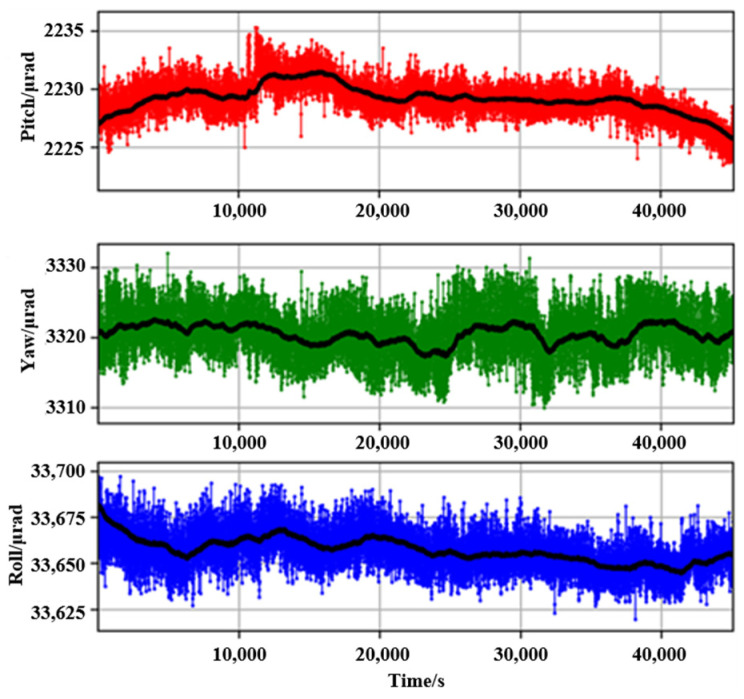
The long-term stability test.

Then, the camera sampling rate was set at the lowest value of 0.5 fps to reduce the video processing time due to the large amount of generated data in the long-term stability test. The original data and the data after 30-point smooth filtering are presented in Figure 11.

The data indicated that the maximum pitch, yaw, and roll drifts within 12.5 h are 11.4 μrad, 22.1 μrad, and 78 μrad, respectively. The noise amplitude after smooth filtering (thick black line) was reduced by more than half. And the stability test results show that the probe has the ability to achieve angle measurement at the microradian level.

### 3.2. Resolution Test

**Figure 12:**
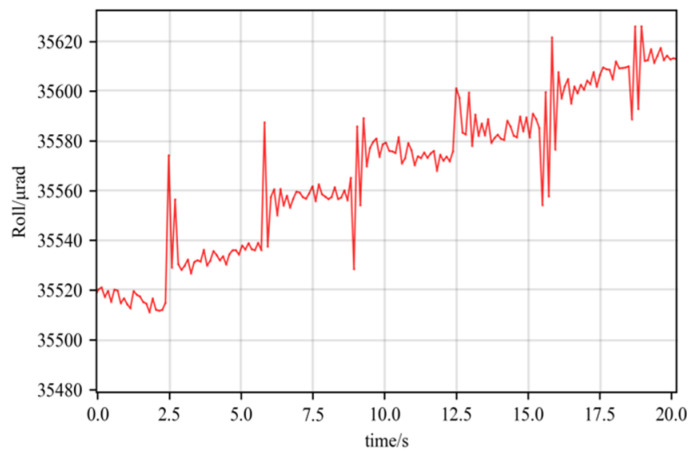
Roll resolution test.

To verify the performance in micro-angle changes of the probe, the combinatorial stage was driven to perform continuous steps around different motion axes. The MGC105 stage was deflected stepwise by 10 μrad to test the roll resolution (Figure 12).

**Figure 13:**
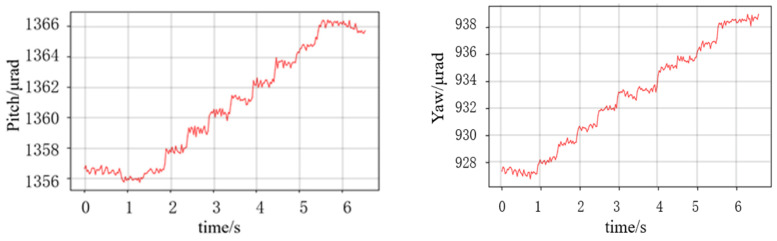
Results of the pitch and yaw resolution.

There is multiple jitter in Figure 12 during and immediately after tilting the table, which was caused by the stage itself. And the roll angle step of 10 μrad can be clearly distinguished if the jitter is not taken into account. Then, the same resolution tests were carried out on pitch and yaw using the P-562.6CD stage. The table was pitch- or yaw-tilted for 1 μrad (Figure 13).

The curve in Figure 13 show that the yaw and pitch resolution of the probe was better than 1 μrad. It demonstrates that the pitch and yaw measurement resolution achieved using a single probe proposed in this paper is comparable to that of similar methods [11,15,16].

### 3.3. Range and Accuracy Test

To verify the measurement error of the probe, measurement range and accuracy tests were carried out. First, the probe measured the angle before and after the deflection of the motion table at a certain angle using an autocollimator as the measurement reference (Table 1).

Notably, the error between the autocollimator and probe measurements in all three dimensions is not greater than 5.1 μrad, indicating that the linear system error of the probe is relatively small.

**Figure 14:**
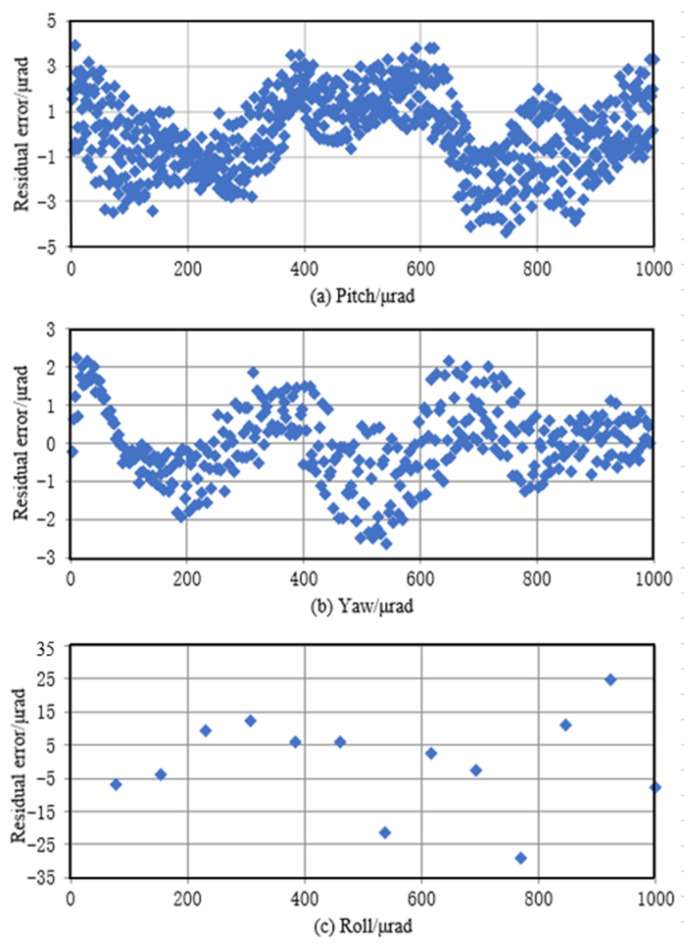
Linear accuracy.

Then, the angle was measured while the motion table was tilted to 1 mrad at a constant speed. As the performance of the motion table controlling the roll change was worse, its minimum speed was faster. However, as the camera sampling frequency is limited to 36 fps, the number of sampling points of the roll was less. Under the constant deflection rate of the motion stage, the angle measured by the goniometer should change linearly. The linear regression analysis of the measured 3-DOF angle data is demonstrated in Figure 14.

The curves show that the roll, yaw, and pitch errors in the range of 1 mrad are below 30 μrad, 4.5 μrad, and 3 μrad, respectively. The probe demonstrates an excellent performance in a wide measurement range at the milliradian level. Compared to a differential wavefront sensing method [14], our method has a larger measurement range and smaller pitch and yaw angle deviation ranges (residual error) but a larger deviation range for the roll angle measurement.

Therefore, tests performed on the shock absorption platform in the clean room demonstrated an enhanced precision of 3-DOF roll, yaw, and pitch angle measurement with a single probe compared to other measurement methods [7,12].

## 4. Discussion

The experiment proved that the developed probe could simultaneously measure pitch, yaw and roll angles. However, the roll resolution is worse than that of pitch and yaw. This can be explained by the difference in the measurement uncertainty formula for these angles.

For a measurement function,

(36) $$y=f(x_{1},x_{2}),$$

its combined measurement uncertainty can be expressed as:

(37) $$u=\sqrt{{\left(\frac{\partial f\left(x_{1},x_{2}\right)}{\partial x_{1}}\right)}^{2}\cdot u_{1}^{2}+{\left(\frac{\partial f\left(x_{1},x_{2}\right)}{\partial x_{2}}\right)}^{2}\cdot u_{2}^{2}+R},$$

where $u_{1}$ and $u_{2}$ are the uncertainties of the two function variables and *R* is the influencing factor that reflects the impact of the correlation between two error terms on the composite uncertainty expressed as:

(38) $$R=2\rho u_{1}u_{2}\frac{\partial f\left(x_{1},x_{2}\right)}{\partial x_{1}}\cdot \frac{\partial f\left(x_{1},x_{2}\right)}{\partial x_{2}},$$

where ρ is the correlation coefficient between the two variables.

Substituting the angle measurement formula, the measurement uncertainty of roll $u_{\alpha }$ and pitch $u_{\beta }$ can be expressed as:

(39) $$u_{\alpha }=\sqrt{{\left(\frac{\lambda }{4n\varphi }\right)}^{2}u_{1}^{2}+{\left(\frac{\lambda }{4n\varphi \cos\varphi }\right)}^{2}u_{2}^{2}+2\rho u_{1}u_{2}{\left(\frac{\lambda }{4n\varphi }\right)}^{2}\frac{1}{\cos\varphi }}$$

(40) $$u_{\beta }=\sqrt{{\left(\frac{\lambda }{4n}\right)}^{2}u_{1}^{2}+{\left(\frac{\lambda }{4n\cos\varphi }\right)}^{2}u_{2}^{2}+2\rho u_{1}u_{2}{\left(\frac{\lambda }{4n}\right)}^{2}\frac{1}{\cos\varphi }}$$

Here, $u_{1}$ and $u_{2}$ are the measurement uncertainty of the left and right fringe frequencies, respectively.

By comparing the two formulas, it can be found that

(41) $$\frac{u_{\alpha }}{u_{\beta }}=\frac{1}{\varphi }\approx 10.18$$

It means that the measurement uncertainty of the roll is ten times larger than that of the pitch.

Another issue is the tradeoff between the measurement resolution of the roll angle and the probe dimensions. The measurement resolution of the roll angle may be improved by increasing the angle φ between two measuring beams. When the angle φ increases, the distance between the reference mirror and the collimator must be increased as well to separate the two measuring beams, thereby enlarging the measuring probe dimensions. Further research will focus on improving the measurement resolution of the roll angle and reducing the probe size by folding the optical path.

## 5. Conclusions

In this paper, a method of simultaneous decoupling of 3-DOF angles based on interference patterns with a single camera is proposed for the first time. A 3-DOF angle probe was developed and a 3-DOF angle decoupling algorithm based on the double interference pattern method was applied to solve the simultaneous measurement problem of pitch, yaw, and roll with a high resolution and stability. The proposed probe construction is extremely simple; it contains only six optical elements and can be easily adjusted. The use of a single measuring mirror and light source is another significant advantage of the probe. In this way, the arrangement and adjustment of the goniometer is simplified and there are no errors caused by inconsistent coordinate references.

The correctness of the measuring principle was verified in a series of experiments, including stability, resolution, and accuracy tests. The performed tests demonstrated that the roll, pitch, and yaw resolutions of the probe are better than 10 μrad, 1 μrad, and 1 μrad, and the errors within the range of 1 mrad are 30 μrad, 3.0 μrad, and 4.5 μrad, respectively. The angle measurement accuracy at the milliradian measurement level meets the vast majority of angle measurement occasions. However, it should be noted that the angle range of the probe needs to be improved, and the accuracy of roll angle still needs compared to those of the pitch and yaw angles.

## Figures and Tables

**Table 1:** Range test statistical results.

Deflecting Direction	Tilt Angle (Measured by Autocollimator)/μrad	Mean Value before Tilting/μrad	Mean Value after Tilting/μrad	Error between Autocollimator and Probe/μrad
Yaw	−1000.0	3516.7	2517.3	0.6
Pitch	998.7	1327.9	2321.5	−5.1
Roll	−998.7	24,918.2	23,892.1	−2.0
Roll	−4998.4	39,104.5	34,133.9	2.8

## Data Availability

Data are contained within the article.
